# National and regional trends in MRI utilization in the face of the ongoing COVID-19 pandemic

**DOI:** 10.1186/s13584-021-00472-y

**Published:** 2021-07-15

**Authors:** Osnat Luxenburg, Mor Saban, Vicki Myers, Sharona Vaknin, Noga Boldor, Rachel Wilf-Miron

**Affiliations:** 1 Medical Technology, Health Information and Research Directorate, Ministry of Health, Jerusalem, Israel; 2 The Gertner Institute for Epidemiology and Health Policy Research, Sheba Medical Center, 1 Emek dotan Street, 5262100 Ramat-Gan, Israel; 3 School of Public Health, Sackler Faculty of Medicine, Tel Aviv University, Tel Aviv, Israel

**Keywords:** COVID-19, Pandemic, Wave, Disparity, MRI exam

## Abstract

**Background and purpose:**

Marked reductions in imaging exams have been documented during the COVID-19 pandemic. The study aimed to examine the effect of the two waves of COVID-19 on magnetic resonance imaging (MRI) utilization at the national and regional level.

**Materials and methods:**

A retrospective-archive study was conducted in Israel, comparing March–December 2020 with March–December 2018 and 2019. Data on MRI utilization were obtained from the national MRI registry, while data on confirmed COVID-19 cases, by place of residence, were obtained from the Israeli Ministry of Health open COVID-19 database.

**Results:**

The number and rate of MRI examinations decreased during the first COVID-19 wave, with the steepest drop in April 2020: 47.5% relative decrease compared to April 2019, and 42.2% compared to 2018. This was followed by a compensatory increase between the waves and a return to almost pre-pandemic levels of use, with just a modest decrease, during the second, more intense COVID wave, compared with the previous year. Existing differences between regions increased during the pandemic. The rate ratio of MRI exams between Tel-Aviv and the Northern periphery increased from 2.89 in April 2019 to 3.94 in April 2020. Jerusalem metropolitan region, with the largest burden of COVID disease, demonstrated only a modest decrease (1%) in MRI utilization during the first 10 months of the pandemic.

**Conclusions:**

At the national level, time trends in reduced MRI utilization followed the first wave of COVID-19, and were accompanied by increased regional disparities. These changes were not explained by differences in the burden of COVID-19 disease but might be explained by unequal distribution of MRI scanners among regions. Reduced utilization was not evident during the second wave, nor at the beginning of the third wave, despite higher COVID-19 case load, demonstrating adaptation to the new normal. Patterns of MRI utilization might help policy-makers and healthcare managers predict the behavior of imaging as well as other sectors, such as elective surgical procedures, during an ongoing pandemic. This forecast might help to manage the lasting effects of the pandemic, including extended waiting times, in the months and years following its remission. In preparation for future national emergencies, timely and detailed data on MRI utilization can serve as a “sensor” for a wide array of diagnostic and interventional medical activities, providing policy-makers with an updated snapshot to guide their response at the regional and national levels.

## Key points

- This study revealed a 47.5% decline in MRI utilization during the first peak of the COVID-19 pandemic, including all exam types, compared with the previous year. This reduction was accompanied by increased regional differences.
- During the second wave of COVID-19, MRI utilization returned to near-normal, despite a higher daily number of new confirmed cases. This describes an adaptation to the “new normal”.
- Patterns of MRI utilization might help forecast the behaviors of other elective as well as non-elective services, for example surgical procedures.
- As the pandemic moves forward, policy-makers can anticipate longer waiting times for MRI, since compensation for decreased utilization during the pandemic was only partial.

## Introduction

The global COVID-19 pandemic has put unprecedented pressure on health care systems, with hospitals and community-based clinics in the worst affected areas close to breaking point. This situation unsurprisingly hampered accessibility and availability of diverse healthcare services [1–3]. In the first wave of viral infections, reductions were documented in most healthcare fields, including elective procedures, screening, emergency department (ED) visits and imaging examinations [4–7]. The American College of Radiology endorsed guidance, issued by the Centers for Disease Control and Prevention, aimed at postponing and rescheduling of non-urgent outpatient visits [8]. This had the greatest impact on screening services, but the effects have been felt throughout the specialty, including interventional procedures. Some radiologists, including trainees, were reassigned to other positions throughout the healthcare continuum, in order to reduce the need for personal protective equipment necessary to care for COVID-19 patients, and limit transmission potential [9]. Others were unable to work due to COVID-19 infection or contact with an infected colleague, since healthcare professionals were at higher risk of being infected, compared with the general population [10].

Unsurprisingly, a significant decline in the volume of radiological activities was observed in the first months of the pandemic across all settings, including EDs and hospital wards [11, 12]. Inpatient volume of imaging activity in a large medical center in New York State, US, decreased by 13.6% during the first 16 weeks of 2020, compared with the parallel period of 2019 [13]. Massive cancellation of non-urgent surgical procedures also resulted in a sudden drop in imaging examinations [12]. For example, an integrated healthcare system serving a population of nearly 3 million people in Ohio, US, reported a larger decrease in outpatient imaging, compared with inpatient imaging – 68 and 31% decrease, respectively [9]. Relative to normal practice, at their lowest point during the 8-week period of March–April 2020, MRI decreased by 56%, and CT decreased by 47% [9]. Another report, from the largest healthcare system in New York State, US, demonstrated the greatest decline in 2020 for outpatient imaging (88%) affecting all modality types, including mammography (94%), MRI (74%) and CT (46%) [13].

Israel is an example of a public healthcare system, with the National Health Insurance Law guaranteeing a predefined list of health services to be provided to all citizens without cost or at nominal charge at the point of service utilization. Healthcare services are provided by four Health Maintenance Organizations (HMOs). MRI is included in the list of health services and provided for a minimal out of pocket sum (quarterly co-payment). In the community setting, where most exams are performed, MRI referrals have to be approved through a pre-authorization system that all HMOs utilize to evaluate the clinical justification of the exam.

In Israel, the first new COVID-19 case was confirmed at the end of February 2020. With rising number of new cases, a national lockdown was imposed for a few weeks in April. In May and June, the number of new cases decreased dramatically, and restrictions were gradually removed. In July 2020, the number of new infections started to rise, reaching a peak in the second half of September, when a second national lockdown was implemented. Public health measures, including the enforcement of mask wearing and social distancing, were the main strategy to tackle the spread of infection throughout these months.

Understanding the association between the health burden of the pandemic (rate of daily new infections, for example) and the utilization of healthcare resources, such as imaging, is critical for policy-makers in order to forecast the surge of imaging activity that will follow declines related to the pandemic. These surges might infer longer waiting times for imaging, delayed diagnosis of urgent health condition and bottlenecks in other healthcare domains that depend on imaging for completion of patient work-up in preparation for elective surgery or initiation of treatment in patients with cancer, to name just a few.

To the best of our knowledge, despite numerous reports on declining volume of imaging exams, including MRI [ 8, 12–15], analysis of the impact on MRI utilization at national level during the first and second COVID-19 waves, has not been published.

### Aim

To examine the effect of the first and second waves of COVID-19 infection on national and regional MRI performance.

## Methods

A retrospective-archive study, conducted in February 2021, reviewed MRI examinations performed in Israel between March and December, comparing 2020 with 2018 and 2019. Data were obtained from the national MRI registry, which includes all MRI examinations performed for both inpatients and outpatients between 2016 and 2021 in all healthcare facilities in Israel and is updated on a monthly basis. All but one of 32 facilities, comprising 97.45% of overall national MRI activity, reported their data in 2020 and were included in the analysis. The registry includes data on the healthcare facility, number of exams performed (adults and children, combined), exam type (anatomic site) and geographic region. The current analysis was conducted at both the national and regional levels and included the following five most common exam types, comprising 91.3% of all MRI examinations in 2019: head, spine, musculoskeletal, chest and abdomen & pelvis. Rate ratio was calculated for the number of exams performed in 2020, compared to previous years (2018 and 2019), overall, by region, and by exam type.

Data on COVID-19 morbidity was collected from the Israeli Ministry of Health (MOH) open COVID-19 database from February 27st (the patient zero case) to December 30th 2020 (10 consecutive months) (https://govextra.gov.il/ministry-of-health/corona/corona-virus) and from the Telegram application- https://t.me/MOHreport). The data covers the whole country, divided into 377 localities, and is updated on a daily basis. The number of confirmed COVID-19 cases was defined as those that tested positive by real-time quantitative reverse-transcriptase polymerase-chain-reaction [qRT-PCR] assay. We used the Central Bureau of Statistics database to allocate each locality from the MOH database to one of the 6 regions (North, Haifa, Tel-Aviv, Center, Jerusalem and South), a commonly used geographic division.

## Results

**Fig. 1:**
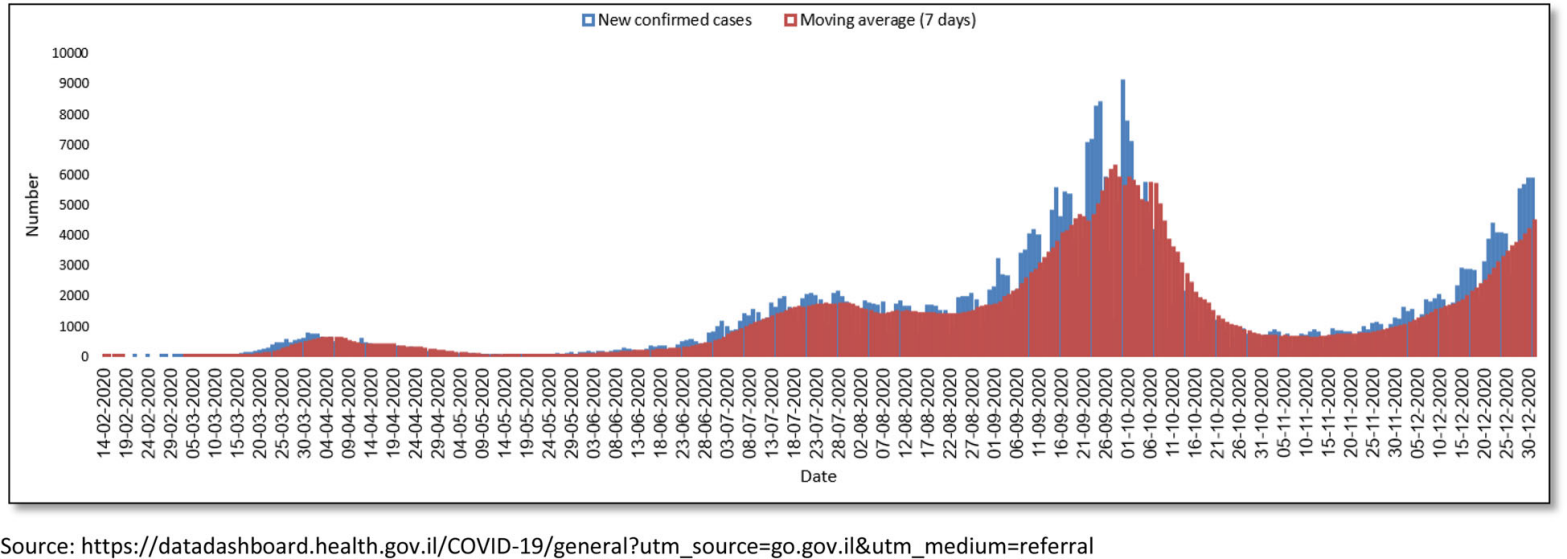
Trends in COVID-19 cases in Israel, February–December 2020

In the 10 months since the first confirmed COVID-19 case, Israel experienced two “waves” or peaks of viral spread, accompanied by national lockdowns: the first reaching a peak in April 2020, and the second gradually increasing from July till a peak in the last week of September and the first week of October 2020. In December 2020, a third wave began (which will not be separately analyzed since our data cover only the first part of this wave). Numbers reached 550 new cases per day at the peak of the first wave, and approximately 7000 new cases per day at the peak of the second wave. Based on the overall population of 9.135 million citizens on 31/12/2019 [14], these figures represent a rate of 6.0 and 76.6 cases per 100,000 population in the first and second waves, respectively (Fig. 1). Between the first and second peaks, there was a period when new cases decreased dramatically, but subsequently began to rise again after many of the restrictions were removed. A similar trend, however less prominent, was the decrease of new cases between the second and third waves.

**Fig. 2:**
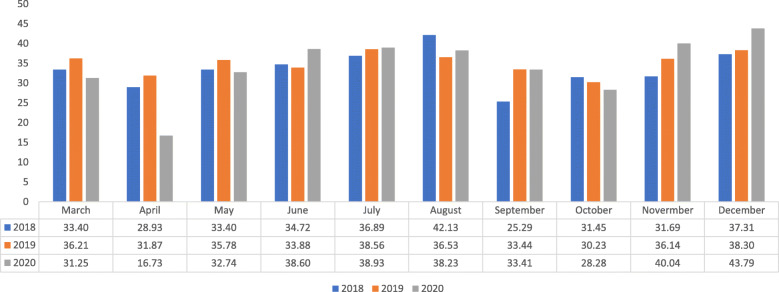
Rates of MRI utilization per 1000 population at the national level during the study period: 2018–2020

The overall number of MRI exams performed during the first 10 months (March–December) of the COVID-19 pandemic in Israel was 342,011, slightly lower compared with the parallel period in 2018 (*n* = 335,196) and 2019 (*n* = 350,909). It should be noted that from 2014 through 2019, the number of exams increased steadily (not shown). In the pandemic months, overall number of exams decreased by 2.5% compared with the same months in the previous year. Figure 2 presents the national rate of MRI exams per 1000 population in March–December 2020, compared to the same period in 2018 and 2019. Exam rate demonstrated the largest reduction in April (0.525 of the activity in the parallel month in 2019, or a relative decrease of 47.5%), with increased performance in June (+ 14.2%) followed by return to pre-pandemic or slightly higher values in July, August and September (+ 1.5%, + 4.7% and + 0.1% respectively) despite the reescalation of COVID cases. In October, which coincided with the highest daily number of new cases, a modest relative decline in exams of 6.5% was noted compared to the previous year. November and December demonstrated a relative increase of 9.1% and 14.3%, respectively.

**Fig. 3:**
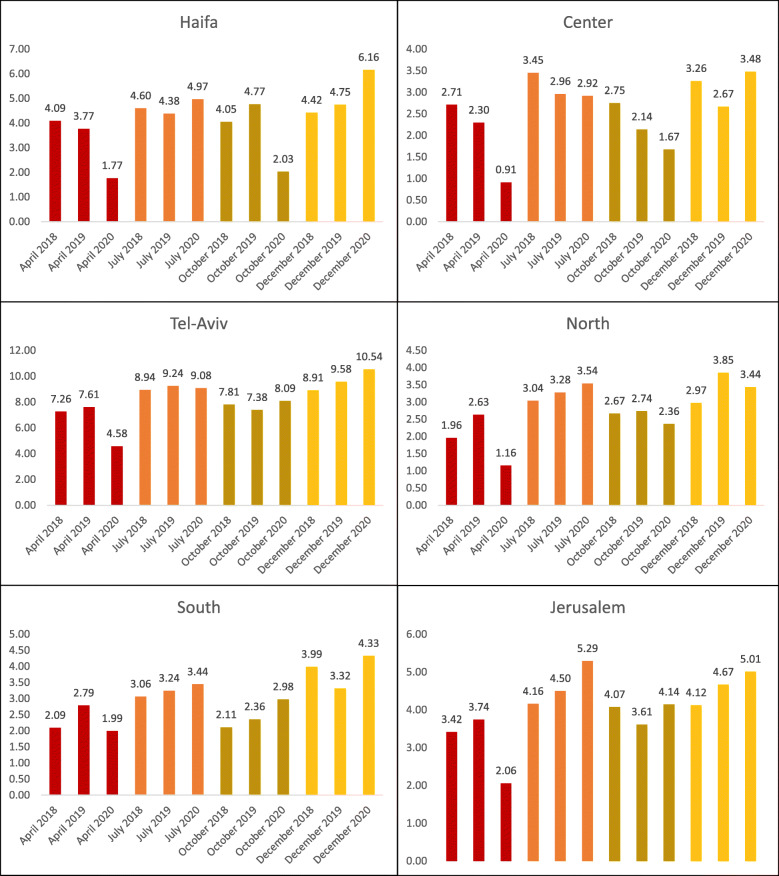
MRI exams per 1000 population, by region. Comparison of 2018–2020 in four selected months

Changes in the rate (number of exams per 1000 population) of MRI performed were analyzed by geographic region, comparing April, July and October 2020 – representing the first (April) and the second pandemic peaks (July–October) with the corresponding months in 2019 (Fig. 3). In April, considerable decrease was documented in all districts. Analysis of the relative change for each region between April 2019 and April 2020, demonstrates the largest decrease in the Central, Northern and Haifa regions − 60.4%, 55.9% and 53.1% relative change, respectively. Differences between regions increased during the first pandemic wave, for example the rate ratio of MRI exams between Tel-Aviv and the Central region increased from 3.31 in April 2019 to 5.03 in April 2020, while the rate ratio between Tel-Aviv and the Northern (peripheral) region increased from 2.89 in April 2019 to 3.94 in April 2020. Other differences, such as between Jerusalem and the Southern (peripheral) region, decreased from 1.34 in April 2019 to 1.03 in April 2020. In both July and October 2020, very small relative changes (both increase and decrease) were noted, compared with 2019. The only exception was Haifa region, which demonstrated a 57.4% relative decline in October 2020, compared with October 2019.

**Fig. 4:**
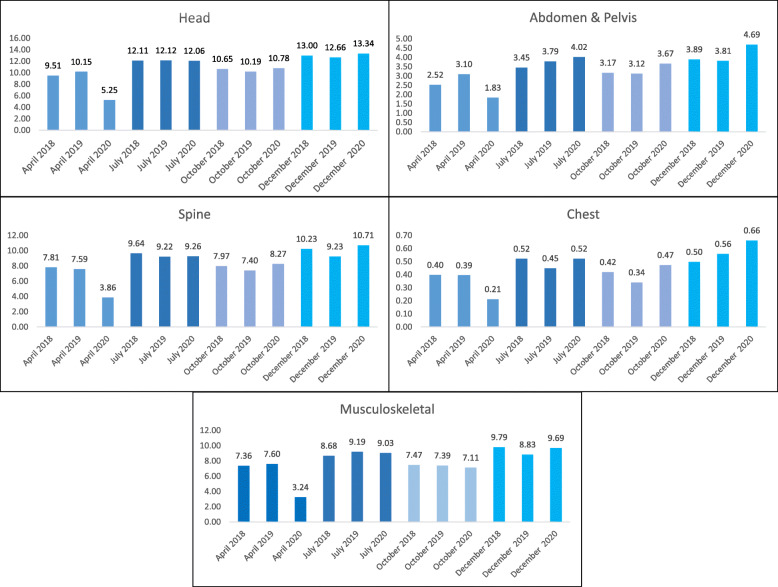
MRI exams per 1000 population by anatomic site: Comparison of 2018–2020 in four selected months

Analysis of MRI exams by anatomic site (Fig. 4) demonstrates decreased performance for all exam types during April 2020, compared with 2019. The maximal relative decline was observed in musculoskeletal MRI (54.1%), followed by head (47.8%), spine (47.6%), chest (46.2%) and abdomen & pelvis (41.4%). In July and October, performance showed minimal change from the previous year (whether slight increase or decrease) for all exam types. The proportion of exams by body part remained stable from 2019 to 2020, with head MRI being most common, followed by spine and musculoskeletal (Fig. 4).

In order to examine a possible association between the burden of COVID-19 disease and changes in MRI performance, we analyzed the difference in MRI exams per 1000 population during the 10-month period (March–December 2020) vs the same period in 2019 and compared it with the rate of confirmed cases per 100,000, by geographic region (Table 1). The burden of confirmed cases in a region was not related to the relative decrease in the volume of MRI activity. Of note, the Haifa region, with a relatively low burden of COVID disease, demonstrated the largest decrease (15%) in MRI performance, while the Jerusalem metropolitan area, with the highest rate of confirmed COVID cases, showed the second smallest relative change in MRIs performed (4% decrease). Examination of the number of MRI scanners per population in the region (a number which remained stable throughout the study period), shows that a relatively high availability of scanners in Jerusalem, was associated with a very modest decrease in MRI utilization despite relatively high burden of disease. Tel-Aviv, however, with the highest MRI distribution, demonstrated a relatively high change in exam performance (11% reduction from the previous year).

**Table 1:** Burden of COVID-19 disease, relative change in MRI exams and distribution of MRI scanners, by region

	Overall	South	Center	Haifa	Jerusalem	Tel-Aviv	North
Confirmed cases per 100 population, March –December 2020	5.11	4.20	4.95	3.99	8.71	4.79	4.66
Rate difference of MRI exams (March–December, 2020/2019)	0.97	1.02	0.96	0.91	0.99	0.97	0.98
Distribution of MRI scanners per million population	5.11	4.72	4.63	4.93	6.31	8.53	4.21

## Discussion

The analysis of time trends in MRI utilization demonstrates a considerable decline during the first pandemic wave, followed by a compensatory increase between the waves and a very modest decrease in the second, much steeper, wave of infections.

During the first months of the pandemic, public health measures were the main defense strategy to reduce transmission, before the emergence of an effective vaccine or medications [15, 16]. In Israel, like other countries, campaigns urged people to “stay at home” and avoid face to face encounters with the healthcare services, giving preference to telemedicine where possible [17]. These messages coincided with governmental orders to stay home when possible. In addition to periods of full or partial quarantine, the fear of being infected at the healthcare facility has discouraged patients from accessing medical services. This resulted in the postponement of diverse diagnostic and therapeutic procedures which might have been perceived as less urgent, such as MRI examination for certain indications, both by the healthcare system and by individual patients. Decreased contact with the healthcare system was noted even among high-risk patients, like women with breast cancer [18].

It should be noted that this trend probably reflects a true decline in clinically justified MRIs, since all referrals for MRI via HMO (most of the exams in Israel) have to be approved based on clinical guidelines, before being accepted in the public healthcare system, though such justification is not currently documented in the national MRI registry. Although there were no official guidelines to postpone MRI in Israel, our study demonstrates a considerable decline in MRI utilization in Israel, in the first but not in the second wave of infections.

Similar to our findings, reductions in imaging have been documented in other countries, though no national studies have been reported to date. Up to 58% decline in imaging volume was documented in a large New York State hospital, parallel to the period when this state was the US epicenter of the pandemic [19]. In a large US health care system, decreased volume of 30% in MRIs was documented for most body regions [13]. The UK NHS reported a 34% decrease in MRI over a similar period [20].

Our study documented a 47.5% relative decline in nationwide MRI performance during April 2020. However, over the following months, utilization gradually returned to normal levels. In June, November and December 2020, a compensatory increase above previous levels was observed, reflecting adaptation to the ongoing pandemic, with intermittent re-opening of most of the country.

What might explain the different MRI utilization patterns between the first and second COVID-19 waves of infection? Why was MRI severely hit by the first but hardly by the second wave? During the first wave, which peaked in April, with an average of 500 new daily cases, strict restrictions were imposed, with a near total lockdown, restricting people to remain within 100 m of their home. Schools were closed for 8 weeks, as was public transport. HMOs urged their members to avoid face-to-face encounters at healthcare facilities and prefer telemedicine where possible. In-hospital activity including emergency department visits and elective surgical procedures dramatically decreased, reflecting the Ministry of Health guidelines to postpone or scale back surgeries that were not considered urgent or time-sensitive [21], and the imposed lockdown, as well as public stress, fear and worry during an unprecedented health disaster of a kind that had never been experienced before. Public compliance with social distancing measures was high: A survey carried out in Israel during February 2020, before the first national lockdown, demonstrated that the public supported compliance with self-quarantine regulations, especially if income would be guaranteed by the government [22].

During the second wave, which peaked in late-September to early October, and was characterized by considerably more cases (7000/day at the peak), less severe restrictions were imposed, including a more “flexible” lockdown for 4 weeks. In the second, longer wave, the public was less compliant with public health orders. Explanatory factors might be the media reports of certain segments within the Israeli society who were reluctant to comply (i.e. certain sectors who kept educational institutions open against the rules, or continued to have large public events (; and the heavy economic toll - an unprecedented unemployment rate (27% in April, 22.7% in October and 13.7% in late December) [23, 24], coupled with the feeling of “beating” COVID-19 after the first wave, since Israel experienced relatively low case fatality rate [25]. Media reports of the toll of delaying care for urgent medical diagnosis and alerts issued by hospital directors and medical associations, urged the public to access healthcare services for any acute morbidity in order to avoid severe complications due to delayed referral. The combination of less strict public health measures, lower public compliance, greater public awareness of the consequences of delaying medical care, and better preparedness of the health care system to care for patients during a pandemic, all contributed to the change in health services utilization. This might explain the return of MRI utilization to normal levels and even surpassing previous levels, as was seen in June–September 2020 [26, 27]. It is likely that the high levels of MRI in June–August and in November–December may have been exams compensating for those delayed from March, April, May and October. Furthermore, the dramatic decrease in elective as well as some urgent surgical procedures during the first wave was followed by a compensatory increase between the waves and during the relatively long second wave (unpublished MOH data). Increased surgical activity might explain part of the pre-operative and peri-operative increased imaging activity, including MRI exams.

The decline that occurred during April 2020 contributed to a widening of existing disparities in MRI utilization between regions. This finding is important, given the pre-existing disparities in the distribution of MRI scanners among regions in Israel. High supply of scanners might explain the fact that despite the highest burden of COVID-19 cases, Jerusalem region was relatively less vulnerable to declining trends in MRI exams.

The relative decline in MRI exams was similar across anatomical sites. This might be explained by the fact that the majority of MRI exams are performed on a non-urgent basis, thus minimizing possible differences in urgency across anatomical sites. MRI is utilized in the ED setting for some urgent indications, such as to identify patients with stroke who are candidates for endovascular intervention [28]. However, despite an increase in ED imaging in recent years, MRI utilization in the ED remains relatively low [29].

The main limitations of the study are: 1) Data on MRI utilization were collected retrospectively, from monthly reports to the national registry, thus precluding a more accurate, real-time analysis of trends. 2) Data were aggregative, which meant that statistical analysis could not be performed to determine the level of significance of the regional and time related differences. The use of aggregative data did not allow analysis of MRI use by patients’ sociodemographic or other characteristics, preventing elucidation of sub-populations at risk for missed exams or change in physician referral patterns. 3) We cannot be certain that the reduced volume of MRI exams represents clinically justified cases, since decision support systems to improve imaging justification are not yet in widespread use in the Israeli healthcare system. 4) We did not have data on other variables which could provide explanations for reduced MRI use, such as quarantined radiology staff or fewer community-based appointments that could lead to requests for diagnostic imaging.

In summary, after a sharp decrease in use during the first wave, the current analysis demonstrated an unexpected return to normal of MRI utilization while COVID-19 infections remained very active, even far more active than in the first wave. The existence of a national MRI registry which collects and analyzes utilization data on a monthly basis, allowed tracking of subtle changes, both at the regional and national levels, caused by the progress of the pandemic.

This study demonstrates the ability of the healthcare system to adapt relatively quickly to the “new normal” reality. At the time of summarizing this study, vaccination of the Israeli population, which began on December 19, 2020, is well underway [30]. However, most public health experts forecast that public health measures will continue at least until the spring of 2021 and some believe that we will all have to adapt to a “new normal” with some social distancing for an even longer period. This highlights the importance of analyzing and understanding the effect of the different stages of the pandemic on health care utilization. The resilience of MRI utilization to a heavy burden of viral infection might reflect a combined effect of the public and the healthcare system. The public - after the initial shock, manifested as great fear of the unknown and unprecedented social distancing - gradually accustomed to the new reality of a pandemic that is here to stay, at least for a while. On the providers’ side, care processes were re-designed, creating strict separation between patients with or without infection. This contributed to reassurance of the public about safety of care and protected staff who were not directly caring for COVID-19 patients. The need for medical providers to resume elective procedures as an important source of income, in face of the heavy medical costs of the pandemic, might be another motive for the increase in MRI activity [31]. These measures might explain the phenomenon demonstrated during the second wave.

### Policy implications

Our study highlights several issues that might be of value to policy-makers in the current era of great uncertainty or might be utilized in future national emergencies:

### MRI utilization as a marker of medical activity trends

The study demonstrated a considerable adaptation of imaging utilization to pre-pandemic levels, despite on-going viral spread. Since MRI supports medical care at different stages, including the diagnosis of new medical conditions, evaluation prior to elective surgeries as well as time sensitive surgeries (such as oncological procedures) [32], our findings might point to changing patterns in the larger phenomenon of healthcare service utilization. Relying on monthly reporting at the national level allows us to elucidate the “adaptation phenomenon”. Other imaging modalities may equally represent changes in medical activity, for example mammography also showed significant reductions during the pandemic [33], however MRI covers a broader range of the population and more clinical domains, making it a suitable proxy.

Our data allows the approximate estimation of when missed imaging exams might be compensated for, and return to pre-pandemic figures, trends which could apply to other medical fields.

The current COVID-19 pandemic has taught us that health disasters can last for a considerable period, in this case for over a year. Meanwhile, delayed elective procedures might become urgent and interrupted diagnosis of new conditions or evaluation of chronic conditions might impose a heavy health toll on patients. It is therefore important to provide policy-makers with a “sensor” that could give an indication, as early as possible, of delays, interruptions and bottlenecks in the provision of healthcare. Israel has a unique tool, the national MRI registry, that provides utilization data on a monthly basis, from all MRI sites throughout the country. In preparation for any future national health emergency, it is advised to collect MRI utilization data on a more frequent (probably weekly) basis, and in more detail, to improve its function as a “sensor’ for a wide array of medical activities.

### MRI as a proxy of the professional and financial toll of the pandemic

Many disciplines have faced substantial challenges during the COVID-19 pandemic. In the imaging discipline, including MRI, reduced activity might interfere with the training of medical students and residents. Similarly, the surgical profession might be influenced by transfer of medical students from clinical care rotations and reduced opportunities for residents to gain experience in the operating room and clinic [34]. The pandemic has also inflicted an unprecedented financial toll on hospitals and HMOs, which when faced with high unexpected costs, have had their existing balance upset. All healthcare organizations, both at the community and hospital levels, will have to invest resources in order to meet the changing health needs of the population.

### Implications for equity in MRI utilization

Our study demonstrated that some differences in MRI utilization between geographic regions increased, while others decreased, during the first peak of the pandemic. These differences are superimposed on existing regional differences in MRI distribution of MRI scanners, as well as differences in MRI utilization. In preparation for the next national emergency, we must better understand those regional, as well as sociodemographic, disparities and explore their response to the current pandemic. This could be done by analyzing MRI utilization by patients’ place of residence, provided by the HMOs as de-codified patient’s ID translated to home address, thus enabling a more accurate analysis of the relationship between neighborhood socio-demographic characteristics and utilization of MRI during and after the pandemic. These data might be enriched by a survey among patients who were referred for a MRI exam during the pandemic, to understand their perception of exam accessibility and availability, reasons for delaying or forgoing care during the pandemic and its effect on their health. This individual and neighborhood level information will allow better understanding of the balance between supply and demand, the latter based on the sociodemographic and health profile of the regions. This, in turn, would allow for mobilization of MRIs (using mobile MRI units) from one area to the other.

Since the pandemic has inflicted a major economic toll on all segments, attention should be given to its long-lasting effects on citizens’ health practices, including screening and non-urgent imaging.

To the best of our knowledge, this is among the earliest national reporting of trends in imaging activity during the second wave. Since MRI is a marker of diverse medical and surgical, elective and urgent activities, it might serve policy-makers, locally and internationally, in forecasting the recovery of healthcare system activity despite an ongoing pandemic. This might allow the monitoring of trends in utilization to better prepare the system’s capacity to deal with the long-term effects of the pandemic including an anticipated “surge” of increased volume, overload and bottlenecks. Further analysis of the factors that influence health service utilization during an on-going disaster may help towards better preparedness for future similar events.

## Conclusions

At the national level, MRI utilization during the COVID-19 waves demonstrated adaptation to the new normal. In preparation for future national emergencies, timely and detailed data on MRI utilization can serve as a “sensor” for a wide array of diagnostic and interventional medical activities, providing policy-makers with an updated snapshot to guide their response at the regional and national levels.

## Data Availability

Data were obtained from the national MRI registry. Data on COVID-19 morbidity was collected from the Israeli Ministry of Health (MOH) open COVID-19 database from February 27st (the patient zero case) to October 30th, 2020 (8 consecutive months) (https://govextra.gov.il/ministry-of-health/corona/corona-virus) and from the Telegram application- https://t.me/MOHreport).

## Abbreviations

HMO: Health Maintenance Organization
ED: Emergency Department
MRI: Magnetic Resonance Imaging
